# Optimization of Screening Strategies for COVID-19: Scoping Review

**DOI:** 10.2196/44349

**Published:** 2024-02-27

**Authors:** Yuanhua Liu, Yun Yin, Michael P Ward, Ke Li, Yue Chen, Mengwei Duan, Paulina P Y Wong, Jie Hong, Jiaqi Huang, Jin Shi, Xuan Zhou, Xi Chen, Jiayao Xu, Rui Yuan, Lingcai Kong, Zhijie Zhang

**Affiliations:** 1 Department of Epidemiology and Health Statistics School of Public Health Fudan University Shanghai China; 2 Key Laboratory of Public Health Safety Ministry of Education Shanghai China; 3 Sydney School of Veterinary Science The University of Sydney NSW Australia; 4 School of Epidemiology and Public Health Faculty of Medicine University of Ottawa Ottawa, ON Canada; 5 Department of Mathematics and Physics North China Electric Power University Baoding China; 6 Science Unit Lingnan University Hong Kong China

**Keywords:** COVID-19, screening strategy, optimization, polymerase chain reaction test, antigen test

## Abstract

**Background:**

COVID-19 screening is an effective nonpharmaceutical intervention for identifying infected individuals and interrupting viral transmission. However, questions have been raised regarding its effectiveness in controlling the spread of novel variants and its high socioeconomic costs. Therefore, the optimization of COVID-19 screening strategies has attracted great attention.

**Objective:**

This review aims to summarize the evidence and provide a reference basis for the optimization of screening strategies for the prevention and control of COVID-19.

**Methods:**

We applied a methodological framework for scoping reviews and the PRISMA-ScR (Preferred Reporting Items for Systematic Reviews and Meta-Analyses Extension for Scoping Reviews) checklist. We conducted a scoping review of the present publications on the optimization of COVID-19 screening strategies. We searched the PubMed, Web of Science, and Elsevier ScienceDirect databases for publications up to December 31, 2022. English publications related to screening and testing strategies for COVID-19 were included. A data-charting form, jointly developed by 2 reviewers, was used for data extraction according to the optimization directions of the screening strategies.

**Results:**

A total of 2770 unique publications were retrieved from the database search, and 95 abstracts were retained for full-text review. There were 62 studies included in the final review. We summarized the results in 4 major aspects: the screening population (people at various risk conditions such as different regions and occupations; 12/62, 19%), the timing of screening (when the target population is tested before travel or during an outbreak; 12/62, 19%), the frequency of screening (appropriate frequencies for outbreak prevention, outbreak response, or community transmission control; 6/62, 10%), and the screening and detection procedure (the choice of individual or pooled detection and optimization of the pooling approach; 35/62, 56%).

**Conclusions:**

This review reveals gaps in the optimization of COVID-19 screening strategies and suggests that a number of factors such as prevalence, screening accuracy, effective allocation of resources, and feasibility of strategies should be carefully considered in the development of future screening strategies.

## Introduction

SARS-CoV-2 has resulted in >500 million cases of COVID-19 worldwide, causing >6 million deaths, and continues to threaten human health [1,2]. The Omicron variant has become the most dominant variant in the current pandemic, and its insidious transmission makes community spread a big challenge [3,4]. For example, the large-scale Omicron outbreak in Shanghai between March and June 2022 resulted in >600,000 infections, and approximately 90% of them were asymptomatic [5]. The global spread of the pandemic has had a tremendous impact on the health of susceptible populations. In China, where the older adult population exceeds 26.4 million [6], the vulnerability of the older adults is heightened, despite the overall low mortality rate of the COVID-19 Omicron subvariant [5,7].

Symptom surveillance and voluntary nucleic acid testing were ineffective in response to the insidious transmission of the Omicron variant. Proactive screening of COVID-19 is essential to identify asymptomatic infections and break the transmission chain in a timely manner [8]. Under the *dynamic zero* policy [9], residents in epidemic areas received nucleic acid screening tests regularly, and the scope and frequency of the screening were dynamically adjusted in accordance with the epidemic trend. Antigen testing is used as a supplement to nucleic acid testing to improve the screening efficiency [10]. COVID-19 screening has become a basic prevention and control measure in countries worldwide, although the scope of screening varies [11]. Different strategies have been developed for the general population [10], international travelers [12], and high-risk populations [13].

COVID-19 screening is the rapid identification of potentially infected individuals by testing a massive population to take appropriate measures, such as isolating the patient, providing treatment, and conducting contact tracing. COVID-19 screening primarily involves nucleic acid and antigen tests. Nucleic acid screening relies on polymerase chain reaction (PCR) testing techniques and is the gold standard for the confirmation of infection [14,15], and the test includes 4 steps: sample collection, preservation, transportation, and testing [10]. The entire process is labor and resource intensive, and each step is important for test accuracy. Antigen screening uses different detection techniques, such as colloidal gold immunochromatography, latex methods, and fluorescence immunochromatography, allowing for quick and easy self-testing. However, antigen testing is less accurate and is often used as a supplement to nucleic acid testing [16,17].

To develop a COVID-19 screening strategy, the target population and the timing and frequency of screening should be considered. For mass nucleic acid testing, a pooled sample testing approach is often used to reduce costs and improve detection efficiency. Factors that influence the cost-effectiveness of outbreak control should also be considered when optimizing screening strategies. In this scoping review of published research, we aimed to summarize the evidence and provide a reference basis for the optimization of screening strategies for the prevention and control of COVID-19.

## Methods

We followed the methodological framework proposed by Arksey and O’Malley [18] and reported according to the PRISMA-ScR (Preferred Reporting Items for Systematic Reviews and Meta-Analyses Extension for Scoping Reviews) [19]. The PRISMA-ScR checklist is available in Multimedia Appendix 1 [19].

### Search Strategy

The search strategy adopted in this review was ((test* OR screen* OR detect*) AND (polymerase chain reaction OR PCR OR nucleic acid OR antigen) AND (COVID* OR SARS-CoV* OR Omicron OR Severe Acute Respiratory Syndrome Coronavirus 2)) NOT (diagno* OR clinic* OR Gene* OR cell OR protein OR laboratory OR patholog*).

The words related to “testing or screening” were limited to the title field. The words related to “testing methods,” “COVID-19,” and “clinical diagnosis or laboratory process technology or pathology” were also limited to the title or abstract fields. PubMed, Web of Science, and Elsevier ScienceDirect were searched for studies published as of December 31, 2022. The reference lists of eligible studies were reviewed to identify additional studies.

### Selection Criteria

The inclusion criteria were as follows: (1) the literature language was English, (2) the disease studied in the publication was COVID-19, and (3) the research articles were related to screening and testing strategies for COVID-19 infection.

The exclusion criteria were as follows: (1) articles not related to COVID-19, (2) duplicates, (3) articles that did not involve screening detection strategies, (4) clinical diagnosis or pathological research articles, (5) technical articles on laboratory testing or testing reagents, (6) environmental detection research articles, and (7) basic theoretical articles on COVID-19 detection techniques. YL and YY screened the literature by reviewing the titles and abstracts. The full-text review was performed by JS, J Hong, KL, and MD, and then, the filtered document was checked again by 1 of these coauthors. Any discrepancies were discussed by YL and YY (Textbox 1).

**Inclusion and exclusion criteria.**

**Inclusion criteria**

- Peer review: Peer-reviewed literature
- Article type: Original articles
- Language: English
- Disease: COVID-19
- Content: Screening and testing strategies for COVID-19 infection

**Exclusion criteria**

- Peer review: Literature not peer reviewed
- Article type: Reviews, meeting articles, comments, and notes
- Language: Non-English
- Disease: Diseases other than COVID-19
- Content: Clinical diagnosis or pathological research; technical articles on laboratory testing or testing reagents; environmental detection research; basic theoretical articles on COVID-19 detection techniques

### Data Abstraction

ZZ, YL, and YY determined which variables to extract, and the latter 2 developed and tested the data-charting form using Microsoft Excel. We abstracted data on the last name of the first author, research design, research population, optimization design, testing method, screening strategy, evaluation index, and recommendation. All authors participated in the data abstraction and reconfirmation of the abstraction. YL and YY charted the data, grouped the studies according to the optimization directions of the screening strategies, and summarized the findings.

## Results

### Overview of Included Studies

**Figure 1:**
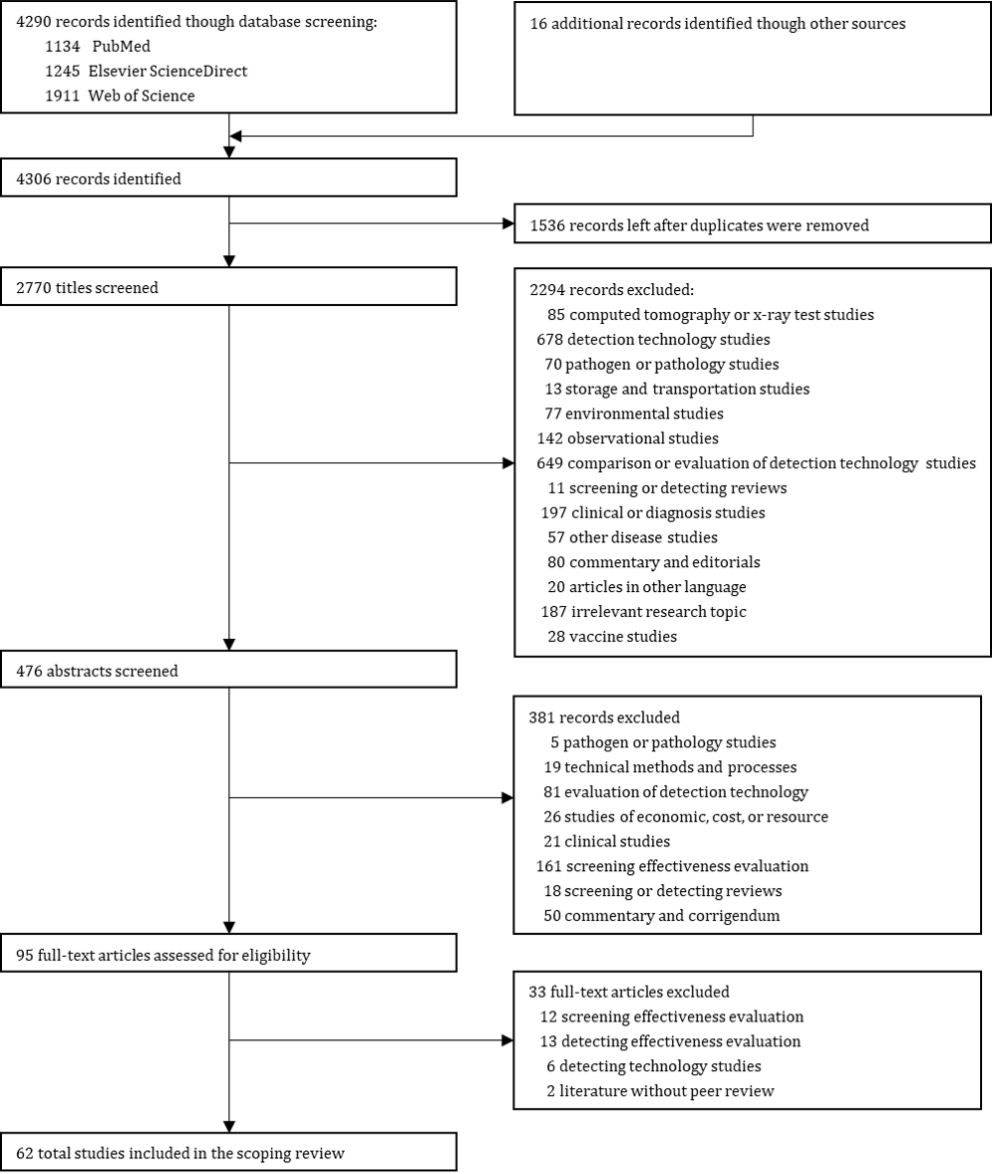
Flow diagram of the search and study selection process following the Preferred Reporting Items for Systematic Reviews and Meta-Analyses Extension for Scoping Reviews.

A total of 4290 publications were found by the searches conducted, of which 1536 were duplicates, and the titles of the remaining 2770 publications were screened for relevance (Figure 1). Subsequently, 476 abstracts were reviewed, and 95 publications received a full-text review. Finally, 62 publications were included in the synthesis (the data-charting form is available in Multimedia Appendix 2 [20-46]).

**Figure 2:**
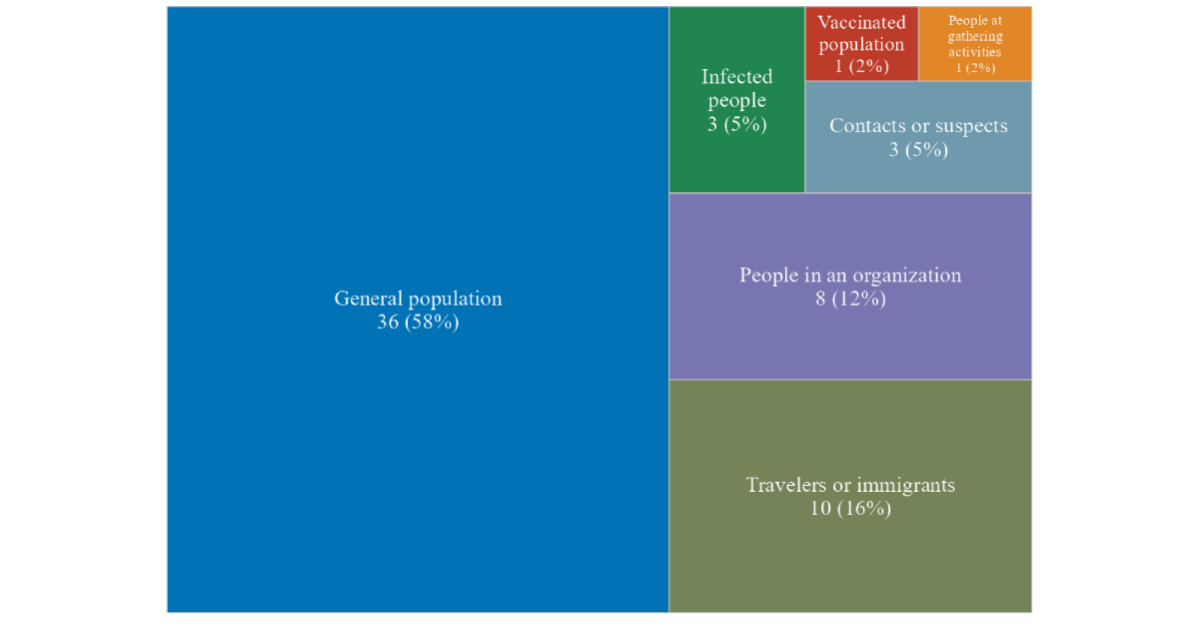
The number of publications on screening population (N=62).

The study populations of the publications were the general population (36/62, 58%), travelers or immigrants (10/62, 16%), people in an organization (8/62, 12%, including workers, health care persons, and students), infected people (3/62, 5%), contacts or suspects (3/62, 5%), vaccinated population (1/62, 2%), and people at gathering activities (1/62, 2%). The optimization designs involved screening the population (12/62, 19%); timing (12/62, 19%); frequency (6/62, 10%); and testing procedure (35/62, 56%), including scenarios for adopting a pooling strategy (10/62, 16%), pool size (19/62, 31%), and pooling approach (18/62, 29%), as shown in Figure 2.

### Optimization of Screening Population

#### Screening of People With Different Infection Risks

Previous studies tried to optimize screening strategies according to infection risk levels indicated by infection probability, contact with an infected person, probability of exposure, or presence of COVID-19 symptoms (Table 1). Du et al [20] simulated COVID-19 testing in populations with different severities of clinical symptoms and age groups and compared infection rates and false-positive rates among groups with different strategies. They found that in settings of high infection rate and limited testing capacity, a greater proportion of tests should be allocated to symptomatic individuals. Conversely, in a surveillance scenario characterized by a low infection rate and sufficient testing capacity, the optimal strategy, which involved directing a higher allocation of tests to people aged >50 years, only required 51.5% of available tests [20]. Likewise, the research by Han et al [21] supported that symptomatic testing at health care facilities was more beneficial than asymptomatic testing in the community, until most symptomatic individuals had been tested. It was also suggested that conducting additional tests to screen for asymptomatic infections among household members yielded the greatest benefit after fulfilling the demand for symptomatic testing [21]. Berestizshevsky et al [22] compared total morbidity, testing efficiency, and infection probability under screening strategies such as no testing and no isolation, symptom monitoring combined with random sampling testing, and symptom monitoring combined with “greedy” testing. Testing and quarantine among symptomatic populations using optimized sampling, which is based on community graphs and population risk factors, results in a 30% to 50% reduction in overall prevalence [22]. Kumar et al [23] compared the effectiveness and cost of outbreak control by weekly reverse transcription-PCR (RT-PCR) testing in 100% antigen-negative vaccinated individuals in a high-income country (the United States) and in a low-income country (India). They suggested that in regions with resource-limited vaccination strategies, high-frequency testing is still needed to minimize subsequent outbreaks [23].

**Table 1:** Research on optimization of COVID-19 screening strategy.

Research population	Optimization directions	Testing methods	Strategy	Evaluation index	Study
General population	Screening population	PCR^a^ and RAT^b^	Optimal testing strategy: allocation of PCR and RAT to different age groups and individuals with varying symptoms while ensuring that that all severe patients are tested and total expenditure remains within the budget Risk‐based strategy Symptom‐based strategy Severe‐only strategy Universal random testing strategy	N_T-pos_^c^, N_T-neg_^d^, N_F-pos_^e^, and N_F-neg_^f^ N_miss_^g^ N_test-pos_^h^ and P_pos_^i^	Du et al [20], 2022
General population	Screening population	RAT	Strategy 1: symptomatic testing at health care facilities Strategy 2: asymptomatic testing in the community setting (households, schools, formal workplaces, or religious gather) with different distribution: (1) even distribution to as many entities as possible once per week and (2) concentrated distribution to test all individuals in selected entities twice a week who will continue to get tested throughout the epidemic. With or without quarantine of household members. 85% of weekly tests for strategy 2 and the rest for strategy 1 All weekly tests for strategy 1 Tests are first used for strategy 1, and any remaining tests are used for strategy 2 next week No testing	Proportion of infections averted relative to the no-testing baseline Number of tests available per 100,000 persons per day Number of additional infections averted for every 100 more tests Reduction of days when R_t_^j^>1 Proportion of infections	Han et al [21], 2022
General population	Screening population	PCR	No testing and quarantining Quarantine people in the state “contagious symptomatic” only Symptom-based plus random or greedy sampling and quarantining of positive people Symptom-based plus sampling based on optimization of community graph and population risk factors and quarantining of positive people	Total and peak morbidity Personal and global quarantine efficiency Number of human-days in different states	Berestizshevsky et al [22], 2021
Vaccinated populations	Screening population	PCR and RAT	RAT followed by PCR testing in 100% of the population weekly in the United States and India	N_infected_^k^ N_cases_^l^ Number of the hospitalized, dead, and recovered Cost	Kumar et al [23], 2022
Travelers	Screening population and screening timing	PCR	PCR testing is not required for travel in all areas PCR testing is required in all regions within 7, 5, and 3 days before travel All those coming from high-risk areas (risk level 3-4) need to be tested within 3 days before travel All those from medium- and high-risk areas (risk level 2-4) need to be tested within 3 days before travel	N_infected_ and N_cases_ N_test_^m^ The medical expenditure	Zhou et al [24], 2021
People at gathering activities	Screening population	PCR	None of the participants are quarantined before the event unless they are contact traced All participants traveling from overseas are quarantined for 14 days before the event All participants are quarantined before the event All mainland participants are tested before the event All participants are tested before the event All participants are tested before the event day 7 following the opening of the event	N_cases_ N_infected_, and percentage change of new and cumulative infections	Wong et al [25], 2022
General population	Screening population	PCR and RAT	Using PCR to test symptomatic patients in outpatient settings Community-based screening by RAT Symptom-driven outpatient diagnostic testing by RAT	Reduction in cumulative symptomatic incidence Number of unnecessary isolations	Baik et al [26], 2022
Workers	Screening population	PCR	No RT-PCR^n^ testing of all workers Testing the workers with COVID-19-like symptoms in isolation Testing the workers without COVID-19-like symptoms but in household quarantine Testing all staff	N_test_ Change of days in quarantine per test Change of workers spreading per test Testing accuracy	Sandmann et al [27], 2020
College students	Screening population	PCR	Testing the students with COVID-19-like symptoms RT-PCR testing for symptomatic students Testing for all students Testing for all students+retesting symptomatic students with a negative first test Testing for all students+retesting all students with a negative first test.	N_T-pos_ and N_T-neg_ N_test_ and N_test_ per person	Van Pelt et al [29], 2021
Travelers	Screening timing	PCR	No measures PCR testing of passengers before embarkation and social isolation PCR testing of passengers before embarkation, daily testing on board, and social isolation	N_cases_	Chowell et al [32], 2021
School students	Screening population	PCR and RAT	Testing based on symptoms and quarantine for 7 days Reactive quarantine of the class level or specialization Reactive screening of the entire class on the day after detection of the case by symptom-based testing, and a screening on days 4 or 7 after case identification Regular testing of the entire school once every 2 weeks or once or twice a week Regular testing with different levels of adherence among the nonvaccinated and reactive closure of the class when every case is detected	R_t_ The proportion of cases reduction N_cases_ Student days lost	Colosi et al [31], 2022
School students	Screening population and screening timing	RAT	Isolation of year group bubbles for 10 days Twice weekly mass testing and isolation of year group bubbles for 10 days Tested daily by RATs for 7 days from the day after identification of every case Twice weekly mass testing and tested daily by RATs for 7 days from the day after identification of every case Twice weekly mass testing No testing or isolation	School days miss per person N_infected_ Asymptomatic cases N_test_ per person Prevalence Absent persons	Leng et al [30], 2022
Health care workers in the nursing home	Screening population	RAT	Testing the health care person within the facility when there are ≥1 positive cases Testing all asymptomatic health care persons in the absence of a known outbreak at predetermined intervals from 1 day to 7 days	Maximum preventable transmission	Zipfel et al [28], 2022
Travelers	Screening timing	PCR and RAT	Testing and quarantine strategies for fully vaccinated travelers and unvaccinated travelers A negative preboarding A negative preboarding test and a negative arrival test Negative preboarding, arrival, and quarantine exit tests 14 days quarantine	R_t_ Adjusted breakthrough IR^o^ Expected number of subsequent infections	Lee et al [37], 2022
Travelers	Screening timing	PCR and RAT	Isolate individuals before or during travel when symptoms appear Test 3 days before travel Test on the day of travel Test 1 day before arrival Test 3 days before arrival The best time to conduct a second test after travel in the absence of postentry quarantine Monitor and isolate symptoms before, during, and after travel 14-day, 10-day, and 7-day isolation	The proportional reduction in transmission risk	Johansson et al [33], 2021
Travelers	Screening timing	PCR, RAT	Predeparture testing No test PCR test 3 days before departure (on day 3) RAT test 1 day before departure (on day 1) Postarrival restrictions Unlimited PCR on days 0 and 4 Daily RAT for 5 days Self-isolation for 5 days with PCR test on days 0 and 4 Self-isolation for 5 days and daily RAT test Government-managed isolation for 7 days and quarantine with PCR test on day 5 Government-managed isolation for 14 days and quarantine with PCR test on days 3 and 12	R_t_/R_0_^p^ The proportion of infected traveler causes, the number of infected travelers that reaches 50 cases from 1 traveler	Steyn et al [34], 2022
Travelers	Screening timing	PCR and RAT	Anterior nose PCR testing within 3 days before departure PCR test within 3 days of departure, on the fifth day after arrival, and isolation for 5 days after arrival RAT within 3 days of departure and on the fifth day after arrival RAT on the day of departure, PCR test on day 5 after arrival, and isolation for 5 days after arrival PCR test on arrival for 5 days	Cumulative infectious days N_infected_ The ratio of N_F-pos_ to N_T-pos_	Kiang et al [35], 2021
Travelers	Screening timing	PCR	RT-PCR tests on arrival and quarantine for 5 days and a second PCR test at the end of quarantine RT-PCR tests on arrival and quarantine for 5 days Quarantine for 14 days without test	IR and proportions of asymptomatic or presymptomatic cases N_miss_ Cumulative probability and hazard rate of developing symptoms	Jen et al [36], 2022
Travelers	Screening timing and screening frequency	PCR and RAT	Isolation only Pretest and inbound testing and isolation Pretest, inbound testing, and outbound isolation and testing Pretest, inbound testing and isolation, and daily testing until the exit Pretest, inbound testing and isolation, and testing every 2 days Pretest, inbound testing and isolation, and testing every 3 days Pretesting, inbound testing, RAT every 3 days, and outbound PCR Pretesting, inbound testing, isolation, and alternative testing at exit (a PCR test or a RAT)	N_miss_	Dickens et al [38], 2021
Contacts	Screening frequency	RAT	Isolation-based strategies: isolation duration of 0, 3, 5, 7, 10, and 14 days after exposure to the case; no testing during isolation or testing on the last day of the isolation period Daily testing strategy: daily RAT of exposed individuals for 1, 3, 5, 7, 10, or 14 days, with no isolation required unless symptomatic or positive testing occurs	Onward transmission potential from secondary cases	Quilty et al [39], 2021
Travelers	Screening timing	PCR	Isolation and no testing Test at the beginning of isolation Test at the end of isolation Test at the beginning and end of isolation Test during the isolating period.	PQTR^q^	Wells et al [40], 2020
Contacts	Screening timing	PCR and RAT	RAT at 2 best times (day 1 and day 3) RAT at 3 best times (day 1 and day 3) and an additional test (PCR or RAT)	The expected number of infection days	Foncea et al [41], 2022
Infected people	Screening timing	PCR and RAT	A RT-PCR test administered 1 or 2 days before the end of quarantine Two RT-PCR tests administered on days 6 or 7 and then on day 8 A 6-day quarantine with tests on days 4, 5, and 6 using a highly sensitive RT-PCR test in cases where the shortest quarantine is needed A RAT with test administered on day 9 or 10 A 9-day quarantine with tests on days 7 and 8	PQTR	Peng et al [42], 2021
General population	Screening frequency	PCR	Citizens, family members, and recent contacts who test positive in the first round of PCR and those who do not participate must be quarantined for 10 days All regions with a positivity rate of ≥0.7% in the first round of testing should undergo a second round of mass testing	The 7-day rolling average of new infections and R_t_	Kahanec et al [43], 2021
General population	Screening frequency	PCR	Community transmission: 2 tests per 1000 people (low incidence) Outbreak response: 4 tests per 1000 people (higher incidence)	N_test_ The percentage of positive tests and the percentage of transmission reduction	Baker et al [45], 2021
Migrant workers	Screening frequency	PCR, RAT	A PCR test every 2 weeks Weekly RAT	R_t_ N_infected_	Koo et al [46], 2022
General population	Screening frequency	RAT	Mass testing with a frequency of fortnightly, weekly, or tridaily testing begins on the 30th day Mass testing with a frequency of fortnightly, weekly, or tridaily testing begins on the peak of the outbreak	N_infected_, N_cases_, and cases of intensive care unit R_t_	Koo et al [44], 2022

^a^PCR: polymerase chain reaction.

^b^RAT: rapid antigen test.

^c^N_T-pos_: number of true-positive results.

^d^N_T-neg_: number of true-negative results.

^e^N_F-pos_: number of false-positive results.

^f^N_F-neg_: number of false-negative results.

^g^N_miss_: number of missed infections.

^h^N_test-pos_: number of people who test positive.

^i^P_pos_: proportion of positive results.

^j^R_t_: effective reproduction number (positives to true positives transmission potential from secondary cases).

^k^N_infected_: number of infected people.

^l^N_cases_: number of confirmed cases.

^m^N_test_: number of tests.

^n^RT-PCR: reverse transcription-polymerase chain reaction.

^o^IR: infection rate.

^p^R_0_: basic reproductive number.

^q^PQTR: postquarantine transmission risk.

#### Screening of People in Different Regions

Table 1 also shows the results of optimizing screening strategies in different regions such as medium-risk or high-risk regions and domestic or foreign regions. Zhou et al [24] compared the total number of infections and daily nucleic acid test loads among the screening strategies and found that the optimal strategy was to test people from medium- and high-risk areas using nucleic acid tests before they traveled. Wong et al [25] assessed infections based on attendance at an event by applying different strategies: no testing and quarantine in all areas, quarantine of attendees from foreign areas, testing of attendees from mainland China, and testing of all attendees at the event. They found that the strategies of quarantining the attendees from foreign areas and testing all (foreign or local) attendees were effective in controlling the number of infections, and they estimated that the total number of new infections was only 1% higher than the current local prevalence [25]. Baik et al [26] simulated the effectiveness of outbreak control in regions with limited resources, such as low- and middle-income countries. They evaluated 3 screening strategies: using PCR to test symptomatic patients in outpatient settings, community-based screening with rapid antigen tests (RATs), and symptom-driven outpatient diagnostic testing using RATs. This showed that RATs would reduce transmission most efficiently when used to test symptomatic individuals in outpatient settings, and to avoid large numbers of unnecessary isolations, mass testing with lateral flow tests (LFTs) should be considered as a screening tool [26].

#### Screening of Occupational Populations and Students

COVID-19 screening strategies were assessed and optimized for some specific student and occupational populations (Table 1). Sandmann et al [27] compared the number of infections, number of tests, and duration of isolation between individuals with and without COVID-19 symptoms living in worker dormitories. Testing all the workers was associated with a reduced transmission of approximately 67 individuals per 1000 tests. However, screening workers with COVID-19-like symptoms in isolation only was associated with a higher risk of transmission in the workplace compared with the strategy of screening all workers [27]. Zipfel et al [28] simulated transmission in health care workers using 2 strategies: testing all when positive cases were detected and testing all periodically at predetermined intervals from 1 day to 7 days. The study showed that 38% of hospital-based transmission could be prevented if all staff were tested within 1 day when a positive case occurred, whereas 30% to 78% of transmission could be prevented if daily testing was performed [28].

Van Pelt et al [29] analyzed the number of RT-PCR tests required to identify each true-positive case and the true-positive rate in a student population using strategies such as symptom screening only, nucleic acid test screening of symptomatic students, and nucleic acid test screening of all students. Conducting RT-PCR testing for all students and retesting those with initially negative results can effectively identify cases with a correct rate of 86.9% [29]. Leng et al [30] further classified the student population by age and simulated the implementation of daily mass screening or screening after the occurrence of positive cases. The study found that mass antigen screening among students significantly reduced the likelihood of not attending class but often required a large number of tests [30]. Similarly, Colosi et al [31] analyzed strategies such as routine testing, symptom-based testing, screening, and quarantine when a positive case was detected and found that weekly testing of 75% of unvaccinated students would reduce the number of cases by 34% in primary schools and 36% in secondary schools.

### Optimization of Timing of Screening

#### Timing of Screening for Travelers

To address the risk of outbreak caused by population movement, researchers compared the timing of screening of traveling people (eg, before, during, and after travel; Table 1). Chowell et al [32] simulated the impact of testing at different times before and after boarding a cruise ship on the cumulative number of infected cases. The study found that testing before boarding, daily testing on board, and maintaining social distancing significantly reduced the possibility of onboard transmission [32]. Johansson et al [33] found that PCR testing on the day of departure and isolation at the destination reduced the risk of transmission. Testing on the day of departure reduced the risk of transmission when traveling by 44% to 72% [33]. Steyn et al [34] assessed the transmission potential of SARS-CoV-2 and the number of infections by simulating PCR or LFT screening at different times, such as the day before departure, the day after arrival at the destination, and the fourth and fifth days after arrival. It was found that the combination of testing and home isolation could reduce the risk of community outbreaks to approximately 0.01, and using daily LFTs or a combination of LFTs and PCR testing could reduce the risk to levels comparable with or lower than those using PCR testing alone [34]. Kiang et al [35] evaluated the cumulative number of days of infection and the number of infections when travelers used PCR or antigen testing at timings such as 3 days before departure, the day of departure, and 5 days after arrival at the destination. The results indicated that nucleic acid testing 3 days before departure reduced the risk of infection during the travel, and the cumulative number of infection days was reduced from 8357 to 5401 days [35]. Jen et al [36] compared the morbidity, missed tests, and proportion of asymptomatic and presymptomatic individuals in travelers with different strategies such as PCR testing on arrival and quarantine for 5 days and quarantine for 14 days without testing. It was found that >82% of the cases would progress from the presymptomatic phase to the symptomatic phase during the 5-day quarantine period, and the quarantine time with 2 PCR tests depended on the risk, testing and quarantine strategy, and vaccination status of the country of departure [36]. On the basis of previous screening strategies for travelers, Lee et al [37] analyzed transmission potential, infection rates, and subsequent infections for strategies such as testing before traveling, after arrival, or at the end of quarantine and 14 days of quarantine without testing, with a consideration of vaccination factors. It was found that at an incidence rate of 0.4 and a time-dependent reproduction number of 16, testing with a sensitivity of ≥98% and specificity of ≥97% both before traveling and on arrival ensured lower expected transmission in vaccinated than unvaccinated individuals with a quarantine of 14 days [37]. For entry-exit pandemic prevention, Dickens et al [38] analyzed the number of unidentified infected persons using the strategies of isolation only; predetection combining entry testing and isolation; and predetection combining entry testing, isolation, and daily testing. The results showed that the risk of transmission was greatly reduced by adopting predetection, which combined entry testing and isolation. During the isolation period, if an RAT was performed every 3 days, only 3% of the infected individuals were unidentified at 7 days and 0.7% at 14 days [38].

#### Screening Timing in Response to an Outbreak

When an outbreak occurs, the appropriate timing of screening facilitates the identification of infected individuals and helps control the spread of the disease. Several studies have explored the impact of different screening timings on the spread of the pandemic (Table 1). Quilty et al [39] compared the impact of daily antigen testing of close contacts over 1, 3, 5, 7, 10, or 14 days on the spread of the epidemic. It showed that quarantining for 7 days with an antigen testing on the last day or daily antigen testing for 5 consecutive days without quarantine was effective in reducing the potential for secondary cases [39]. Wells et al [40] evaluated the impact of screening timing, such as at the start or end of isolation and during the period of isolation, on the risk of continued transmission after isolation. It was suggested that PCR testing at the start and end of isolation could reduce the risk of continuous transmission and shorten the isolation period from 14 days to 7 days. However, testing only at the start of isolation had no notable effect on reducing the risk of transmission and shortening the isolation period [40]. Foncea et al [41] simulated the screening of close contacts at different timing, such as days 1, 2, and 3 after exposure to an infector, and compared the expected days of infection during outbreaks. This suggested that antigen testing should be performed on days 1 and 3 for epidemic prevention and control. Two tests were sufficient to effectively prevent infection, and the effectiveness was equivalent to a 14-day isolation period when personnel compliance was 80% to 90%. If an additional test (PCR or antigen) was performed, it was equivalent to the 14-day isolation period effectiveness when personnel compliance was 90% to 100% [41]. Peng et al [42] conducted a similar study to assess the risk of spread after the end of the quarantine period for PCR testing at different timings, such as the days 1, 2, 4, 5 and 6 during the quarantine period. The results showed that PCR or antigen testing at different timings reduced the quarantine period to different degrees without increasing the risk of transmission. Combining testing with shorter quarantine periods is more cost-effective in terms of both time and expenses compared with a 14-day quarantine. For instance, using 3 highly sensitive RT-PCR tests along with a 6-day quarantine yielded a similar risk of transmission as the traditional 14-day quarantine. [42].

### Optimization of Screening Frequency

Screening frequency should maximize the effectiveness of screening testing and minimize the related costs. Studies on the impact of different screening frequencies on COVID-19 control in communities or high-density populations are also presented in Table 1. Kahanec et al [43] found that 14 days after 2 rounds of mass nucleic acid testing, the infection rate decreased by approximately 30% and the basic reproductive number decreased by approximately 0.3. In a simulation study, Koo et al [44] found that the influence of the test frequency was greater than the maximum test sensitivity (range 0.6-0.8) on the number of infections. The average reduction in infections per day between the 2 testing days was 2.2%, whereas each 1% increase in test frequency reduced infections by an average of 0.43% [44]. Baker et al [45] found that if the number of screening tests per day was slightly higher than the daily testing capacity, it would not cause a burden on testing, but more cases could be found and transmission could be reduced more effectively. Koo et al [46] assessed the impact of biweekly PCR tests or weekly RATs on the number of new infections and infectivity in areas with a high population density (such as workers’ dormitories) and found that biweekly PCR testing (39 new cases per month) was as effective as weekly RATs (33 new cases per month) and could prevent local outbreaks.

### Optimization of Screening Procedure

#### The Importance of the Implementation of the Pooling Strategy

Given the huge demand for sampling and testing, it is important to optimize the screening procedure, and the pooling strategy (ie, collecting multiple samples in a pool for testing) has been frequently used. For example, the National Health Commission of China and the Centers for Disease Control and Prevention of America have issued guidelines for pooled sample tests [47,48]. Three issues related to the pooling strategy have been studied: scenarios for adopting the pooling strategy, pool size, and test procedure.

#### Scenarios for Adopting a Pooling Strategy

In general, the decision to adopt a pooling strategy is determined by comparing the average number of pool tests per person with a baseline number of 1 (Multimedia Appendix 3 [49-83]). A study demonstrated that when the prevalence exceeded 0.1, the average number of tests per person was >1 for pooled testing with a pool size of 32; such pooled testing was no better than the individual test [49]. The average number of tests per person varied with the pool size. Choosing an optimal pool size can minimize the average number of tests per person. In this case, when the prevalence was <0.07, the pooling strategy could save the need to perform more than half of the tests. When the prevalence was close to or >0.3, the number of pooled tests was close to or exceeded that of the individual test [50]. A prevalence of 0.3 may be considered as the threshold for performing pooled testing.

#### Pool Size

The optimal pool size can be calculated based on expected positive rate and detection accuracy with the objective of minimizing the number of tests, and the number of tests decreases with decreasing prevalence and increasing pool size. If the accuracy was 100% and the prevalence was 0.001, 0.005, or 0.01, the optimal pool sizes were estimated to be 32, 15, and 10, respectively [51]. There is a certain upper threshold value for the pool size that is limited by testing accuracy (Multimedia Appendix 3).

To estimate the optimal pool size, the prevalence (positivity rate or infection rate) must be assumed. It is also assumed that all individuals are independent of each other, and that the probability of infection is uniform. However, in practice, the prevalence remains unknown until the test results are available (Multimedia Appendix 3). Pikovski and Bentele [52] considered the prevalence to be a random variable uniformly distributed between the expected maximum and minimum values substituted in the calculation of the optimal pool size. An optimal pool size of 4, 3, or 5 was acceptable when the prevalence was uniformly distributed between 0 and 0.3 [52]. In addition, there is heterogeneity and correlation in the probability of infection among people. Fewer tests are needed when individuals in the same pool for testing are homogeneous in terms of age, sex, and other risk characteristics [53-55]. Libin et al [56] considered that combining pools with several families for testing was more conducive to home isolation. The propagation dynamics simulation found that a family-based pool size of 32 and testing volume of 50,000 per day could achieve the weekly testing of the entire population in Belgium [56]. A larger optimal pool size is required considering the correlation of individuals in the pool [57], or even the social graph [58], in which an edge represents frequent social contacts between 2 persons. Furthermore, Augenblick et al [59] showed that if the pool size could be adjusted to be optimal with the infection rate at any time, screening with a high testing frequency could quickly reduce the infection rate. The final number of tests may decrease despite the high testing frequency owing to the increasing optimal pool size, that is, “frequency gain” [59].

Test accuracy, including screening sensitivity and specificity, also affects the optimal pool size and the upper limit of the pool size. Bish et al [60] found that the optimal pool size would modestly increase when the sensitivity of the pooled sample test decreased. In the PCR test, the sensitivity would decrease and the specificity would increase in a pooled sample test owing to the dilution effect [61], which needs to be considered in the calculation of the optimal pool size [62]. The maximum pool size recommended in previous studies varies from 8 to 30 [63-65]. The sensitivity of the individual test and the influence of the dilution effect of pooling on sensitivity are related to the specific techniques of sampling and testing (such as sampling tools, sample processing reagents, detection instruments, and standardization of operation); therefore, test accuracy is an important determinant for the selection of pool size.

#### Pooling Approach

For the original Dorfman pooling approach, each individual in a positive pool is tested separately. If a pooled sample is negative, then all individuals in the pool are regarded as negative. Several suggestions were made to optimize the pooling approach, which are summarized in Table 2.

First, sequential pooling may be used. A positive pool is divided into several subpools, and the samples in the positive subpools are tested individually [55,66]. Binary pooling divides people to be screened into 2 pools, and the positive pool is divided and pooled again until all positive individuals are found [67]. In the nested pooling strategy, the samples in a positive pool are divided into smaller pools with an optimal number of stages, and the optimal pool size of each stage is calculated according to various measures such as the predicted number of positives and time limit [68,69]. Ng et al [70] conducted simulations of a household-based sequential pooling approach to optimize a universal testing scheme in Hong Kong. They showed that the household-based sequential pooling approach could rapidly screen people in high-risk groups for COVID-19 infections and quarantine those who tested positive [70]. Although these approaches reduce the number of tests required, the operability of such strategies for time-critical epidemic control should be carefully assessed.

Second, repeated testing of the same pool of samples may be conducted to reduce false negatives of the pooled test [71]. Litvak et al [72] conducted a second pooled test after reordering and recombining the samples in the negative pools. For the sequential pooled test, some researchers allocated a part of the samples to 2 subpools to improve accuracy [73].

Third, a copy-link optimization strategy may be used to accurately link the results of the pooled test to the individuals in the pool. The primary “copy-link” strategy is matrix pooling [84]. Samples are arranged in the form of a matrix, with each row and column forming a pool, and each sample is tested once in the row pool and once in the column pool. Research on matrix pooling for COVID-19 has only mathematically simulated the number of tests and the accuracy of the pooled sample test. The Dorfman pool test may be more economical when the prevalence is extremely low, whereas matrix pooling may be more economical when the prevalence is relatively high [74]. Žilinskas et al [75] broadened the concept of matrices by dividing each sample into 2 pools to create as many links as possible between pools. Zhou and Zhou [76] applied the copy-link strategy in designing the Pentagram minipool test. Mutesa et al [77] expanded the 2D matrix to a 3D or multidimensional hypercube, where the number of copies of each sample was split into different planar slices of the hypercube. The subsamples on 1 planar slice of the hypercube were tested in a pool together. Investigators have demonstrated the feasibility of this “hypercube testing strategy” in the laboratory, and field trials are underway in Rwanda and South Africa [77]. Wu et al [78] improved the current hypercube testing strategy by calculating the prevalence, edge, and dimension because every edge had a best performance range, and hypercube pooling with edge=3 may not be the optimal strategy in different outbreaks. Daon et al [79] used a Bayesian model to determine the best combination of pool size, detection steps, repeat detection, and split sample detection to maximize the mutual information between the infection status and testing results. However, this is limited to a simulation analysis.

**Table 2:** Research on optimization of pooling approach in COVID-19 screening strategy.

Strategy	Evaluation index	Study
Dorfman pool test Pool test for each sample tested in multiple pools	Daily detection capacity N_test-saving_^a^ Sensitivity Number of sample results represented by each test	Cleary et al [80], 2021
Individual test Pool test repeated multiple times	The number of persons per test The upper bound for the fraction of N_miss_^b^ FNR^c^	Hanel and Thurner [71], 2020
Individual test Dorfman pool test Splitting pool test: samples in the negative pools are recombined to new pool tests, and samples with 2 negative results are identified as negative	N_F-pos_^d^ N_test_^e^ N_F-neg_^f^	Litvak et al [72], 2020
Dorfman pool test Sequential pool test: the positive pool is divided into several subpools of pool size of 3, and the samples in the positive subpool are tested individually	N_test_ Ratio of number of tests	Cheng et al [66], 2021
Random sequential pooling test the positive pool is divided into several subpools, and the samples in the positive subpool are tested individually Informed sequential pool test: divide subjects with similar risk of infection (eg, by age and sex into the same pool)	N_test_ per person	Millioni and Mortarino [55], 2020
Individual test Binary pool test of the best number and depth of branches considering the prevalence	Ratio of number of tests	Perivolaropoulos and Vlacha [67], 2021
Nested pool test: the positive pool is then divided into several small pools	N_test_ per stage N_test_ per person Accuracy Cost N_cases_^g^	Armendáriz et al [68], 2021
Pool test strategy based on the optimization algorithm: the positive pool is then divided into several small pools and tested in the next stage	The percentage of tests required compared with individual testing	Rai et al [69], 2020
Pooling test of pooling size of 20 in the family with different prevalence and then retest with minipool for batches with positive results using pooling size of 20, 10, 5, 4, and 2	N_test_	Ng et al [70], 2022
Multistage pool test: ≥3 stages using the overlap strategy (some samples are detected in both pools)	IR^h^ N_test_	Gu et al [73], 2021
Individual test Dorfman pool test Matrix pool test	Costs Cost per test Positive rate	Kim et al [74], 2022
Individual test Matrix pool test OptReplica pool test: each patient is allocated in the first pool and replicated in another pool with the smallest number of allocated patients	N_test-saving_	Žilinskas et al [75], 2021
Individual test Dorfman pool test Pentagram minipool test: for the positive Dorfman pooling with size of 10, double samples are tested by 5 “three-in-one” pools and 1 “five-in-one” pool	N_test_	Zhou and Zhou [76], 2022
Subsample pool test in the hypercube algorithm	Loss of sensitivity compared with individual test N_test_ per person and N_infected_^i^	Mutesa et al [77], 2021
Pooling test under different prevalence, edge, and dimension using the hypercubic method	N_test_	Wu et al [78], 2022
Dorfman pool test Recursive pool test Matrix pool test D-Optimal Pool Experimental design (a novel Bayesian pooling strategy)	FNR and FPR^j^ N_test_	Daon et al [79], 2021
Individual test 2-stage Dorfman pool test Binary splitting pool test Optimized recursive binary splitting pool test Matrix pool test Sobel-R1: a decision tree approach based on binomial distribution	Confirmed cases per test Time to test the whole population N_T-pos_, N_F-pos_ N_cases_ Number of quarantined individuals	de Wolff et al [81], 2020

^a^N_test-saving_: number of saving tests compared with individual testing.

^b^N_miss_: number of missed infections.

^c^FNR: false-negative rate.

^d^N_F-pos_: number of false-positive results.

^e^N_test_: number of tests.

^f^N_F-neg_: number of false-negative results.

^g^N_cases_: number of confirmed cases.

^h^IR: infection rate.

^i^N_infected_: number of infected people.

^j^FPR: false-positive rate.

^k^N_T-pos_: number of false-negative results.

## Discussion

### Principal Findings

Despite ongoing vaccinations worldwide, COVID-19 is still present and causing outbreaks, and screening remains important. First, SARS-CoV-2 has the potential to mutate, and the transmissibility, pathogenicity, and incubation period of the novel variant remain unknown. Screening facilitates the surveillance and tracking of SARS-CoV-2 novel variants, providing essential information for an appropriate response. In addition, screening helps protect vulnerable populations and reduce the pressure on the health care system. Therefore, the continuous optimization of screening strategies to improve cost-effectiveness and reduce resource consumption is still worthy of our attention in the current global situation of relaxed prevention and control. Furthermore, the development of strategies for screening COVID-19 provides a basis for the prevention and control of novel or re-emerging infectious diseases in the future, particularly respiratory infectious diseases.

In previous studies, researchers have been optimizing the screening strategy for COVID-19 based on the target population, timing, frequency of screening, and testing procedure and providing a scientific basis for COVID-19 screening. Specific strategies are designed and developed for different populations according to risk levels, regions, or occupations; different timing and frequencies (eg, before, during, and after traveling or entry and exit); and different testing procedures (eg, individual or pooled test, pool size, and polling approaches). The conceptual model for developing screening strategies is available in Multimedia Appendix 4. As there is a continuous emergence of new variants of SARS-CoV-2, further research is necessary to improve the current screening strategy by addressing the issues on the scale of screening, proper timing and frequency of testing, testing accuracy, and cost-effectiveness.

### Dynamic Adjustment of Screening Strategies Based on Variations in Prevalence

The design and optimization of any screening strategy are based on disease prevalence during an epidemic as a hypothetical condition. This determines the strictness of the screening strategy, including the screening frequency, time interval, target population, and testing procedures. For example, the selection of pool size in pooled testing depends on the prior prevalence. To apply the optimization plan in practice, the information on real prevalence is important for determining the screening plan at a specific time in a specific area, which may be obtained from the screening results at the same time. Nicholson et al [85], Hamadeh et al [86], and Chiu and Ndeffo-Mbah [87] explicitly estimated the real prevalence by statistically correcting the reported data, which can be used in the research and practice of optimizing and adjusting screening strategies over time. Additionally, pandemics are constantly evolving. Screening results can help policy makers and researchers understand current risk levels and trends as well as reflect past screening effects. A feedback mechanism should be created to dynamically adjust the screening strategy according to the screening results. When the number of cases increases rapidly, the stringency of screening should be increased to prevent a larger pandemic outbreak. For example, Yu et al [82] set several alternative pool sizes and updated the pool size within an alternative range every week according to the changes in prevalence in a simulation study. A dynamic screening plan should be relatively stable, easy to implement, and adaptable to the changing trend of an epidemic or outbreak.

### Optimizing Screening Strategies by Combining Multiple Testing Methods

Various testing methods have been developed. Some methods are more appropriate than others for different screening processes. Self-detection, such as the use of antigen tests, has been widely promoted. As a supplement to laboratory detection methods, such as PCR, it can effectively save the resources of medical workers for sampling and testing. It has been applied for the screening of both small and large populations. However, the accuracy of the current antigen test is lower than that of the PCR test, with an overall sensitivity of approximately 70% and a specificity of approximately 98%, and its accuracy for asymptomatic patients is lower than that for symptomatic patients [88]. Moreover, there are no guaranteed standardization of sampling for self-detection and compliance with self-isolation after diagnosis. Self-detection is often an alternative when professional testing capabilities are insufficient, and self-detection and laboratory testing complement each other. A few studies have compared laboratory tests with self-tests or combined antigen tests with PCR tests [35,38,39,42,46,86]. Yu et al [82] found that the PCR pooled test tended to be more cost-effective at low prevalence because it allows more people to be tested with existing equipment and quarantines more patients with presymptomatic and asymptomatic infections to prevent future infections. However, a pooled PCR test would cause delays in results and would not facilitate timely quarantine of infected persons and interrupt transmission at a high prevalence. In contrast, high-frequency antigen screening may reverse the epidemic to obtain results quickly and quarantine infected people in a timely manner, despite the large number of false positives in the screening process [82]. Although the sensitivity of the PCR pooled test may be higher than that of the RAT, there is a problem with a large number of tests leading to delays in reporting. RATs are particularly useful in settings such as schools, workplaces, and mass gatherings with a high frequency owing to their characteristics of lower cost, rapid time to result, and increased accessibility. A combination of laboratory testing and self-detection may maximize the benefit under limited resources, which needs to be studied in the future.

### Accuracy of Screening Test

Whether an infected person can be identified depends on their infection status after exposure because viral nucleic acid, antigen, antibody, and other biomarkers change with infection time. The accuracy of testing results may also change with time. These characteristics can help in the selection of the appropriate timing for screening and quarantine of close contacts and entry-exit persons with a definite exposure time [39].

Screening accuracy is affected by sampling quality and detection accuracy. In China, sampling quality is monitored based on whether human somatic cells are collected from the swab as a laboratory indicator [89]. As the dilution effect caused by pooling is likely to reduce test sensitivity, it is crucial to consider PCR testing with optimal sensitivity and the maximum pool size. The sensitivity may be affected by the sample handling method, selection of the detection kit, and standardization of the detection operation [90]. The accuracy of screening should be evaluated when designing pooling strategies, which is conducive to the selection of pool size and the credibility of the screening results.

### Optimization of PCR Testing Procedures to Improve Detection Capacity

Optimizing the nucleic acid detection procedure for the pooled sample test using various approaches reduces the number of tests performed and increases the detection speed and capacity, which can improve the speed of obtaining results for quarantine-infected individuals. The primary Dorfman procedure has been implemented during COVID-19 screening in some areas owing to its convenience and operability. The pool size when screening the entire population in China was 5, 10, or 20 [48,91], and the US Food and Drug Administration authorized 5 pool tests with pool sizes ranging from 3 to 10 [47]. The optimal selection and dynamic adjustment scheme of the pool size need to be in accordance with the actual prevalence and detection accuracy. The pooling procedure has been improved using a multistage strategy and “copy-link” strategies for higher detection efficiency [81]. The multistage strategy reduces the number of tests while simultaneously increasing the number of test rounds. The turnaround time from sampling to result reporting may increase, but its operability has not yet been investigated. The “copy-link” strategy not only reduces the amount of testing but also requires only 1 testing stage to locate positive individuals. However, for this strategy, the laboratory requires more complex testing procedures and equipment. It is not feasible to apply “copy-link” strategy to an existing laboratory platform.

### Accurately Define the Scope of Screening Individuals

A screening strategy for the whole population can effectively control the outbreak, but it also has a significant impact on health care resources and delays the time of transferring cases and contacts to quarantine [45]. Therefore, the occurrence of cases does not imply the need for screening the entire population. In China’s *dynamic zero* policy, screening of the entire population of an area is often initiated in the early stage of an outbreak and interrupts the spread of COVID-19 [92]. However, there is a lack of research on when and under what circumstances a full screening should be initiated, and whether specific thresholds for the number of cases or incidence are necessary in the decision-making process. High-risk populations are usually targeted for screening such as people from high-risk areas or countries or people with special occupations. Screening helps to isolate infectious sources to prevent COVID-19 outbreaks [93].

### Consider the Implementability of Screening Strategies

Previous studies on the optimization of screening strategies have mainly focused on how to control an epidemic, reduce the risk of transmission, and shorten the isolation period. However, the design and implementation of screening strategies depend on the resources available, which vary greatly among countries or regions. A large-scale screening program is costly and may not only be sustainable owing to limited resources.

Larremore et al [94] raised the meaningful problem of whether the frequency of testing, time to obtaining results, or sensitivity is more important in new crown screening. Their study revealed that effective testing depends largely on the frequency of testing and the speed of obtaining results, whereas the sensitivity of the test is relatively secondary [94]. This means that the implementation of relatively low-sensitivity PCR pooled tests or antigenic tests can improve the speed of obtaining results, facilitate quarantine of infected individuals, and interrupt transmission. This may be appropriate in areas with high prevalence.

### Methods of Comparing Different Screening Strategy

When searching for an appropriate screening strategy, the most commonly used method involved using model simulations to evaluate the effectiveness of different screening strategies. For the selection of models to compare different screening strategies, the appropriate model should be based on different situations, populations, and purposes. In addition, real-world disease data are always available during the pandemic, but very few studies have evaluated the effectiveness of different screening strategies from the perspective of real-world data. The conclusions of evaluating the effectiveness of screening strategies using real-world data are more realistic. Model simulations enable a convenient comparison of the effectiveness of different screening strategies for different situations, but they are based on a hypothetical theoretical setting. Furthermore, if the results from the model simulations can be validated using real-world data, the findings of these studies will be more reliable.

Regarding the comparison of different screening strategies, which is the best strategy depends on the actual situation. The selection of a screening strategy in realistic scenarios requires a balanced consideration of the economic costs and effectiveness of controlling the outbreak. Only a few studies reviewed in this paper analyzed cost-effectiveness in a cursory manner; for example, the simple indexes such as cost per test [74] or cost per infection reduced [82] were used. Furthermore, attention should also be paid to resource consumption, such as medical resources (eg, hospital beds) and quarantine resources (eg, hotel rooms), which determine the feasibility and sustainability of the strategy [95].

### Limitations

First, we restricted studies to those publications in English and did not search gray literature and might have missed relevant studies published in other languages and in non-peer-reviewed journals and conference proceedings. Second, we restricted the search terms to the title or abstract field and might have excluded some studies that included the search terms as Medical Subject Headings terms or free text. Third, we did not perform duplicate screening of publications owing to time limitations. However, data extraction was performed by 9 investigators from our team, and 2 investigators in cooperation conducted quality assessments (YL and YY) to ensure consistency. Finally, most publications in this review were simulation studies using mathematical models, which lack accepted criteria for quality evaluation; therefore, literature quality assessments were not performed.

### Conclusions

A well-designed and developed COVID-19 screening strategy is conducive to the rapid identification of infected individuals and the control of an epidemic. As new variants continue to emerge, screening strategies should be dynamically adjusted and optimized to achieve expected results. To identify and isolate infected individuals in a timely manner, a screening strategy must produce fast and accurate results. A program is sustainable only when costs can be controlled at the level of available resources. Some key elements for COVID-19 screening strategies are reviewed and discussed, including the screening population, timing and frequency of screening, detection methods, and procedures.

## Appendix

Multimedia Appendix 1 PRISMA-ScR (Preferred Reporting Items for Systematic Reviews and Meta-Analyses extension for Scoping Reviews) checklist.

Multimedia Appendix 2 Research on the optimization of COVID-19 screening strategy.

Multimedia Appendix 3 Research on the optimization of SARS-CoV-2 nucleic acid detection strategy.

Multimedia Appendix 4 Conceptual model for screening strategy development.

## Abbreviations

LFT: lateral flow test
PCR: polymerase chain reaction
PRISMA-ScR: Preferred Reporting Items for Systematic Reviews and Meta-Analyses extension for Scoping Reviews
RAT: rapid antigen test
RT-PCR: reverse transcription-polymerase chain reaction

## Data Availability

All data generated or analyzed during this study are included in this published article and its supplementary information files.
