# Supplementary material for: Optimization of Screening Strategies for COVID-19: Scoping Review
Source: JMIR Public Health Surveill. 2024 Feb 27;10:e44349. doi: 10.2196/44349 (PMC10933748; doi:10.2196/44349)
Supplement: Multimedia Appendix 2 [file publichealth_v10i1e44349_app2.docx]

**Multimedia Appendix 2**

Table 1. Research on optimization of SARS-CoV-2 screening strategy.

| Research design | Research subjects | Optimization directions | Testing methods | Strategies | Evaluation index | Recommendations | References |
| --- | --- | --- | --- | --- | --- | --- | --- |
|  |  |  |  |  |  |  |  |
| Simulation study | General population | Screening population | PCR^a^, RAT^b^ | A. Optimal testing strategy: allocation of PCR and RAT to different age groups and individuals with varying symptoms while ensuring that that all severe patients are tested total and expenditure remains within the budget.  B. risk‐based strategy  C. symptom‐based strategy  D. severe‐only strategy  E. universal random testing strategy | ·N_T-pos_^c^  ·N_T-neg_^d^  ·N_F-pos_^e^  ·N_F-neg_^f^  ·N_miss_^g^  ·N_test-pos_^h^  ·P_pos_^i^ | ·Detecting mode: In high prevalence settings with limited number of tests, the majority of tests are allocated to symptomatic people, and he majority of asymptomatic testing are allocated to young people.  ·Surveillance mode: Allocated more tests to asymptomatic and old people in low prevalence settings with adequate testing resources. | Du et al [20], 2022 |
| Simulation study | General population | Screening population | RAT | Strategy 1: symptomatic testing at healthcare facilities  Strategy 2: asymptomatic testing in the community setting (households, schools, formal workplaces, or religious gather) with different distribution: 1) even distribution to as many entities as possible once per week, and 2) concentrated distribution to test all individuals in selected entities twice a week who will continue to get tested throughout the epidemic. With or without quarantine of household members.  A. 85% of weekly tests for Strategy 2, and the rest for Strategy 1.  B. All weekly tests for Strategy 1.  C. Tests are first used for Strategy 1, and any remaining tests used for Strategy 2 next week.  D. No testing. | ·Proportion of infections averted relative to the no-testing baseline  ·Number of tests available per 100000 persons per day  ·Number of additional infections averted for every 100 more tests  ·Reduction of days when R_t_ ^j^> 1.  ·Proportion of infections. | ·Strategy 1 yields greater benefits than any Strategy 2 until most symptomatic individuals who sought testing have been tested.  ·The demand of Strategy 1 is highly influenced by individuals without SARS-CoV-2 but other respiratory tract infections.  ·After the demand of Strategy 1 is satisfied, excess tests to proactively screen for asymptomatic infections among household members yield the largest additional infections averted. | Han et al [21], 2022 |
| Simulation study | General population | Screening population | PCR | A. No testing and quarantining.  B. Quarantine people in the state "contagious symptomatic" only.  C. Symptom-based plus random sampling and quarantining of positive people.  D. Symptom-based plus greedy sampling and quarantining of positive people.  E. Symptom-based plus sampling based on optimization of community graph and population risk factors, and quarantining of positive people. | ·Total and peak morbidity  ·Personal and global quarantine efficiency.  ·Number of human-days.  during which the person is (i) healthy and free; (ii) contagious and quarantined; (iii) healthy and quarantined; (iv) contagious and free. | The community-graph models the interactions between people, and can help determine the infection risk in individuals and perform the investigations of “chains-of contagion”. | Berestizshevsky et al [22], 2021 |
| Simulation study | The vaccinated populations | Screening population | PCR, RAT | RAT followed by PCR testing 100% of the population weekly in the United State and India. | ·N_infected,_  ·N_cases,_  ·Number of the hospitalized, dead, and recovered  ·Cost | A resource-limited vaccination strategy still requires high-frequency testing to minimize subsequent outbreaks. | Kumar et al [23], 2022 |
| Simulation study | Travelers | Screening population and screening timing | PCR | A. PCR testing is not required for travel in all areas.  B–D. PCR testing is required in all regions within 7/5/3 days before travel.  E. All those coming from high-risk areas (risk level 3–4) need to be tested within 3 days before travel.  F. All those from medium and high-risk areas (risk level 2–4) need to be tested within 3 days before travel. | ·N_infected_^k^ relative reduction of N_infected_  ·N_cases_^l^ (total, exported, imported, and locally transmitted)  ·N_test_^m^  ·The overall medical expenditure | ·Pre-travel testing could reduce the number of active infections.  ·Testing passengers from risk tier 2–4 regions 3 days before travelling could significantly reduce the risk of transmission, and it is more economical and efficient than testing for all passengers. | Zhou et al [24], 2021 |
| Simulation study | People at gathering activities | Screening population | PCR | A. None of the participants are quarantined before the event unless they are contact traced;  B. All participants travelling from overseas are quarantined for 14 days before the event.  C. All participants are quarantined before the event.  D. All mainland participants are tested before the event.  E. All participants are tested before the event;  F. E + day 7 following the opening of the event. | ·N_cases_  ·N_infected_ (new and cumulative), percentage change of new/cumulative infections | Universal testing for participants is likely to be an effective and feasible intervention. | Wong et al [25], 2022 |
| Simulation study | General population | Screening population | PCR, RAT | A. Using PCR to testing symptomatic patients in outpatient settings.  B. Community-based screening by RAT.  C. Symptom-driven outpatient diagnostic testing by RAT. | ·Reduction in cumulative symptomatic incidence  ·Number of unnecessary isolations | ·RAT would reduce transmission most efficiently when used to test symptomatic individuals in outpatient settings.  ·To avoid large numbers of unnecessary isolations, mass testing with LFAs should be considered as a screening tool. | Baik et al [26], 2022 |
| Simulation study | Workers | Screening population | PCR | A. No RT-PCR testing of all workers.  B. Testing the workers with COVID-19–like symptoms in isolation.  C. Testing the workers without COVID-19–like symptoms but in household quarantine  D. Testing all staff. | ·Infection level  ·N_test_  ·Days in quarantine  ·Workers spreading  ·Change of days in  ·Quarantine per test change of workers spreading per test  ·Testing accuracy | It is recommended that testing staff in household quarantine or all staff depending on infection levels and testing capacities. | Sandmann et al [27], 2020 |
| Simulation study | College students | Screening population | PCR | A. Testing the students with COVID-19-like symptoms.  B. RT-PCR testing for symptomatic students.  C. Testing for all students.  D. C + retesting symptomatic students with a negative first test.  E. C + retesting all students with a negative first test. | ·N_T-pos_  ·N_T-neg_  ·N_test_  ·N_test_ per subject | Given the cost of RT-PCR testing, a staged approach could be adopted: initial testing of all students, followed by a decision on repeat testing based on infection prevalence. | Van Pelt et al [29], 2021 |
| Simulation study | Travelers | Screening timing | PCR | A. No measures.  B. PCR testing of passengers prior to embarkation and social isolation.  C. PCR testing of passengers prior to embarkation, daily testing on board and social isolation. | ·N_cases_ | The combination of PCR testing before boarding, daily testing after boarding, and social distancing can greatly mitigate the number of cases during cruises. | Chowell et al [32], 2021 |
| Simulation study | School students | Screening population | PCR, RAT | A. Testing based on symptoms and quarantine for 7 days.  B. Reactive quarantine of the class level or specialization.  C. Reactive screening of the entire class on the day after detection of the case by symptom-based testing, and a screening on days 4 or 7 after case identification.  D. Regular testing of the entire school once every 2 weeks or once or twice a week.  E. Regular testing with different levels of adherence among the non-vaccinated and reactive closure of the class when every case detected. | ·R_t_  ·The proportion of cases reduction  ·N_cases_  ·Student days lost | Extending vaccination coverage in students, complemented by regular testing with good adherence, are essential steps to keep schools open when highly transmissible variants are circulating. | Colosi et al [31], 2022 |
| Simulation study | School students | Screening population and screening timing | RAT | A. Isolation of year group bubbles for 10 days.  B. Twice weekly mass testing and strategy A.  C. Tested daily by RATs for 7 days from the day after identification of every case.  D. Twice weekly mass testing and strategy C.  E. Twice weekly mass testing.  F. No testing or isolation. | ·School days miss per subject  ·N_infected_  ·Asymptomatic cases  ·N_test_ per subject  ·Prevalence  ·Absent subjects | ·It highlights the conflict between the goals of minimizing within-school transmission, minimizing absences, and minimizing testing burden. But it did not identify one that minimized all three aspects simultaneously.  ·An assessment of the relative benefits and costs of each factor must be made when considering public health policies. | Leng et al [30], 2022 |
| Simulation study | Healthcare workers in the nursing home | Screening population | RAT | A. Testing the healthcare person within the facility when one or more positive cases.  B. Testing all asymptomatic healthcare persons in the absence of a known outbreak at predetermined intervals from 1 to 7 days. | ·Maximum preventable transmission | Periodic testing would need to be implemented with high frequency and sensitivity. | Zipfel et al [28], 2022 |
| Simulation study | Travelers | Screening timing | PCR, RAT | Testing and quarantine strategies for fully vaccinated travelers and unvaccinated travelers:  A. A negative preboarding.  B. A negative preboarding test and a negative arrival test.  C. Negative preboarding, arrival, and quarantine exit tests.  D. 14 days quarantine. | ·R_t_  ·Adjusted breakthrough infection rate  ·Expected number of subsequent infections | In most cases, fully vaccinated travelers (with or without booster) and a negative preboarding test can be released with a negative rapid antigen test upon arrival. | Lee et al [37], 2022 |
| Simulation study | Travelers | Screening timing | PCR, RAT | A. Isolate individuals before or during travel when symptoms appear.  B. Test 3 days before travel.  C. Test on the day of travel.  D. Test one day before arrival.  E. Test 3 days before arrival.  F. The best time to conduct a second test after travel in the absence of post-entry quarantine.  G. Monitor and isolate symptoms before, during and after travel.  H. 14-day, 10-day, and 7-day isolation. | ·The proportional reduction in  transmission risk | The measures, including symptom monitoring, testing, and quarantining, can be more effective when used together, and the optimal test timing depends on the effectiveness of quarantine. | Johansson et al [33], 2021 |
| Simulation study | Travelers | Screening timing | PCR, RAT | Pre-departure testing:  A. No test.  B. PCR test 3 days before departure (on day 3).  C. RAT test 1 day before departure;(on day 1).  Post-arrival restrictions:  D. Unlimited.  E. PCR on days 0 and 4.  F. Daily RAT for 5 days.  G. 5 days self-isolation with PCR test on days 0 and 4.  H. Self-isolation for 5 days and daily RAT test.  I. 7 days government-managed isolation and quarantine with PCR test on day 5.  J. 14 days government-managed isolation and quarantine with PCR test on days 3 and 12. | ·R_t_/R_0_^n^  ·The proportion of infected traveler causes, the number of infected travelers that reaches  50 cases from one traveler | Using daily RAT or a combination of RAT and PCR tests can reduce risk to a comparable or lower level than using PCR tests alone. Combined with controls on the number of travellers from countries with high prevalence, there are different options for managing the risk of COVID-19 at the border. | Steyn et al [34], 2022 |
| Simulation study | Travelers | Screening timing | PCR, RAT | A. Anterior nose PCR testing within 3 days prior to departure.  B. PCR test within 3 days of departure, on the 5th day after arrival and isolation for 5 days after arrival.  C. RAT within 3 days of departure and on the 5th day after arrival.  D. RAT on the day of departure, PCR test on day 5 after arrival, and isolation for 5 days after arrival.  E. PCR test on arrival for 5 days. | ·Cumulative infectious days  ·N_infected_  ·The ratio of N_F-pos_ to N_T-pos_ | Routine asymptomatic testing before travel can be an effective strategy to reduce the risk of infection during travel, although abbreviated quarantine with post-travel testing is probably needed when travelling from a high to low incidence setting. | Kiang et al [35], 2021 |
| Simulation study | Travelers | Screening timing | PCR | A. RT-PCR tests on arrival and quarantine for 5 days and a second PCR test at the end of quarantine.  B. RT-PCR tests on arrival and quarantine for five days.  C. Quarantine for 14 days without test. | ·Daily incidence rate  ·Proportions of asymptomatic or pre-symptomatic cases  ·N_miss_  ·Quarantine days  ·Cumulative probability of developing symptoms  ·Hazard rate of developing symptoms | The time required for quarantine given two PCR tests depends on the risk of departing countries, testing, and quarantining strategies, and whether the passengers have vaccine jabs. | Jen et al [36], 2022 |
| Lab study and simulation Study | Travelers | Screening timing and screening frequency | PCR, RAT | A. Isolation only.  B. Pre-test and inbound testing and isolation.  C. Pre-test, inbound testing, and outbound isolation and testing  D. B and daily testing until the exit.  E. B and testing every 2 days.  F. B and testing every 3 days.  G. Pre-testing, inbound testing, RAT every 3 days, and outbound PCR.  H. Pre-testing, inbound testing, isolation, and alternative testing at exit (a PCR test or a RAT). | ·N_miss_ | Mandatory testing is conducted at pre-testing and upon arrival, with the isolation time determined by reference to the risk level of the destination country, pandemic preparedness and the origin of the traveler. | Dickens et al [38], 2021 |
| Lab study and simulation study | Contacts | Screening frequency | RAT | A. Isolation-based strategies:  Isolation duration of 0, 3, 5, 7, 10, and 14 days after exposure to the case, no testing during isolation or testing on the last day of the isolation period.  B. Daily testing strategy: Daily RAT of exposed individuals for 1, 3, 5, 7, 10, or 14 days, with no isolation required unless symptomatic or positive testing occurs. | ·Onward transmission potential from secondary cases | A higher focus on detection and a reduction in the time required to reach secondary cases may avoid a greater proportion of transmission, but it may require a proportional extension of the quarantine period or the number of days of detection. | Quilty et al [39], 2021 |
| Lab study and real-world study | Travelers | Screening timing | PCR | A. Isolation and no testing.  B. Test at the beginning of isolation.  C. Test at the end of isolation.  D. Test at the beginning and end of isolation.  E. In the case of isolation from 1 to 14 days, the test is conducted on the Nth day respectively. | ·PQTR^o^ | Properly timed testing can shorten isolation times and address the false negatives that occur at high prevalence rates. | Wells et al [40], 2020 |
| Simulation study | Contacts | Screening timing | PCR, RAT | A. Two best time (day 1 and day 3) RAT.  B. A and an additional test (PCR or RAT). | ·The expected number of infection days | Simple testing rules can be effective for improving contact tracing in settings where strict quarantine adherence is difficult to implement. | Foncea et al [41], 2022 |
| Simulation study | Infected people | Screening timing | PCR, RAT | A. A RT-PCR test administered 1 or 2 days before the end of quarantine.  B. Two RT-PCR tests administered on days 6 or 7 and then day 8.  C. A 6-day quarantine with tests on days 4, 5, and 6 using a highly sensitive RT-PCR test in cases where the shortest quarantine is needed.  D. A RAT with test administered on day 9 or 10.  E. A 9-day quarantine with tests on days 7 and 8. | ·PQTR | Testing reduces the length of isolation and the physical and mental stress caused by lengthy quarantines. Test- assisted quarantines could be safer and more cost-effective than 14-day quarantines. | Peng et al [42], 2021 |
| Simulation study | General population | Screening frequency | PCR | A. Citizens, family members and recent contacts who test positive in the first round of PCR and those who do not participate must be quarantined for 10 days.  B. All regions with a positivity rate of 0.7% or higher in the first round of testing should undergo a second round of mass testing. | ·The 7-day rolling average of new infections, R_t_ | Mass testing and quarantines could be an effective tool and re-testing at regular intervals may be necessary. The impact of repeated mass antigen testing on reducing new infections peaked approximately two weeks after round 2 and gradually faded out thereafter. | Kahanec et al [43], 2021 |
| Simulation study | General population | Screening frequency | PCR | A. Community transmission: 2 tests per 1000 people (low incidence).  B. Outbreak response: 4 tests per 1,000 people (higher incidence). | ·N_test_  ·The percentage of positive tests, the percentage of transmission reduction | ·Asymptomatic patients are prioritized for testing, and the order of close contacts and symptomatic patients depends on the initial outbreak or community transmission.  ·It causes delays if daily testing exceeds the routine capacity. Those delays can undermine efforts of contact tracing and quarantine. It is recommended that the testing volume be slightly higher than the routine testing capacity. | Baker et al [45], 2021 |
| Simulation study | Migrant workers | Screening frequency | PCR, RAT | A. A PCR test every 2 weeks.  B. Weekly RAT. | ·R_t_  ·N_infected_ | Mass PCR testing or weekly rapid antigen testing in high population density environments is needed and repeated mass testing is highly effective for preventing localized site outbreaks. | Koo et al [46], 2022 |
| Simulation study | General population | Screening frequency | RAT | A. Mass testing with a frequency of fortnightly, weekly, or tridaily testing begins on the 30th day.  B. Mass testing with a frequency of fortnightly, weekly, or tridaily testing begins on the peak of the outbreak. | ·N_infected_ (daily, cumulative)  ·N_cases_ (daily, cumulative)  ·R_t_  ·N_cases_ of ICU | Large-scale use of RAT mitigates the impact and impact of COVID-19 on the healthcare system, even if the sensitivity is lower than the PCR. Regular mass testing of the population is therefore a possible strategy that effectively intervenes in countries transitioning from pandemic mitigation to endemic COVID-19. | Koo et al [44], 2022 |

^a^PCR: Polymerase chain reaction.

^b^RAT: Rapid antigen test.

^c^N_T-pos_: Number of true positive results.

^d^N_T-neg_: Number of true negative results.

^e^N_F-pos_: Number of false positive results.

^f^N_F-neg_: Number of false negative results.

^g^N_miss_: Number of missed infections.

^h^N_test-pos_: Number of people who test positive.

^i^P_pos_: Proportion of positive results.

^j^R_t_: Effective reproduction number (positives to true positives transmission potential from secondary cases).

^k^N_infected_: Number of infected people.

^l^N_cases_: Number of confirmed cases.

^m^N_test_: Number of tests.

^n^R_0_: Basic reproductive number.

^o^PQTR: Post quarantine transmission risk.
