# Supplementary material for: Optimization of Screening Strategies for COVID-19: Scoping Review
Source: JMIR Public Health Surveill. 2024 Feb 27;10:e44349. doi: 10.2196/44349 (PMC10933748; doi:10.2196/44349)
Supplement: Multimedia Appendix 3 [file publichealth_v10i1e44349_app3.docx]

**Multimedia Appendix 3**

Table 1. Research on optimization of SARS-CoV-2 nucleic acid detection strategy.

| Research design | Research subjects | Optimization direction | Testing methods | Strategies | Evaluation index | Recommendations | References |
| --- | --- | --- | --- | --- | --- | --- | --- |
|  |  |  |  |  |  |  |  |
| Simulation study | General population | Whether pooling and pooling size | PCR^a^ | A. Individual test.  B. Pooling test of several pooling sizes considering the prevalence. | ·N_test_^b^ per subject  ·N_test_ | When the prevalence is below 0.1, pooling test is worth considering. | Bilder CR, et al. (2021)[49] |
| Simulation study | General population | Whether pooling and pooling size | PCR | A. Individual test.  B. Pooling test of several pooling sizes considering the prevalence. | ·Minimum N_test_ per subject to diagnose one case  ·N_add_^c^ of positive for every 100 tests  ·Subjects tested in 100 tests using pooling test with optimal pooling size | ·The pooling test works best in settings with a low prevalence.  ·It is best implemented in subgroups with low clinical suspicion. | Aragón-Caqueo D, et al. (2020)[50] |
| Simulation study | General population | Whether pooling and pooling size | PCR | A. Individual test.  B. Pooling test of several pooling sizes considering the prevalence. | ·N_test_ per subject | At high target prevalence (>0.1), pooling test can marginally improve testing capacities, whereas pooling at rather low target prevalence may substantially enhance sample throughput. | Regen F, et al. (2020)[51] |
| Simulation study | General population | Whether pooling and pooling size | PCR | A. Individual test.  B. Pooling test of the optimal pooling size with sensitivity of 0.71/0.98/1 considering the prevalence. | ·N_test_ per subject  ·N_test-saving_^d^ | The testing throughput of pooling test with weekly updates of pool sizes is higher than individual tests. To achieve the maximum benefits of pooling test, it is important to use pooling sizes that are customized for different risk groups in the population, and designed according to the prevalence dynamically and uncertainly. | Bish DR, et al (2021)[60] |
| Simulation study | People in the businesses or school | Whether pooling and pooling size | PCR | Pooling test of the optimal pooling size considering the prevalence with or without a confirmatory round. | ·N_test_ per subject  ·N_test-saving_  ·Specificity and sensitivity | ·Reopening businesses and schools will require reliable testing data.  ·Pooling test could lower testing costs, especially in low-prevalence areas. | Lakdawalla D, et al. (2020)[61] |
| Lab study | People in the healthcare | Pooling size | PCR | Pooling test of pooling size of 2, 4, 8, 16, 32, and 64 (RT-PCR). | ·Ct value | ·Pooling test can be applied immediately in the current clinical testing laboratories.  ·Implementation of a pool test for COVID-19 allows expanding current screening capacities thereby enabling the expansion of detection in the community, as well as in close organic groups. | Yelin I, et al. (2020)[83] |
| Lab study | Asymptomatic cases | Pooling size | PCR | Pooling test of pooling size of 4–30 (RT-PCR). | ·Ct value | ·Pooling of up to 30 samples per pool can increase test capacity and detects positive samples with sufficient diagnostic accuracy.  ·Borderline positive single samples might escape detection in large pools. | Lohse S, et al (2020)[63] |
| Lab study and  real-word study | Suspected positive people | Pooling size | PCR | Pooling test of pooling size of 4, 8, 16, and 32. | ·Ct value  ·Specificity and sensitivity  ·F-measure = 2/(1/specificity+1/sensitivity) | A 1/16 dilution of a positive sample is a practical limit. The pooling strategy is recommended in countries with low potential screening infrastructures. | Bensaada M, et al. (2022)[64] |
| Lab study, simulation study and real-world study | Population with low viral load | Pooling size | PCR | Pooling test of pooling size of 5–15 (RT-PCR). | ·Ct value | The pooling size needs modification as the pandemic progresses. Prevalence should be carefully estimated before pooling tests are conducted. | Jeong H, et al. (2021)[65] |
| Simulation study | General population | Whether pooling, pooling size and pooling approach | PCR, RAT^e^ | A. Individual PCR test (60 days for the whole population).  B. Pooling PCR test of the optimal pooling size which is weekly updated.  C. Antigen test (every 3 days).  D. Antigen test (every 14 days). | ·Sensitivity  ·N_test_ per case  ·IR (cumulative, over time)  ·Number of screening rounds  ·N_F-neg_^f^, N_F-pos_^g^  ·Negative predictive value, positive predictive value  ·Cost per infection reduced  ·End time of the epidemic | ·PCR‑based pooled screening is cost‑effective in reversing the pandemic at low prevalence, when the prevalence is high, may not stop the outbreak.  ·Antigen screening with sufficient frequency could reverse the epidemic, despite the high cost and the large numbers of false positives in the screening process. | Yu, J, et al. (2021)[82] |
| Simulation study | People in the skilled nursing facility | Pooling size | PCR | A. Individual test.  B. Pooling test of several pooling sizes especially 4 and 10.  C. Pooling test of the optimal pooling size. | ·N_test_  ·N_test-saving_  ·Cost  ·Saving cost compared to individual test  ·N_F-neg_ | ·Pooled testing is an efficient strategy for congregate settings with a low prevalence of COVID‐19.  ·Dilution as a result of pooling can lead to erroneous false‐negative results. | Nianogo RA, et al. (2021)[62] |
| Simulation study | General population | Pooling size | PCR | Pooling test of the optimal pooling size considering the minimal and maximal prevalence. | ·Ratio of number of tests  ·FPR^h^ | ·A pool size of four is recommended.  ·Testing errors reduce the sensitivity and increase the specificity compared to a test without pooling. | Pikovski A, et al. (2020)[52] |
| Simulation study | General population | Pooling size | PCR | A. Dorfman pooling test.  B. Pooling test with specific pooling sizes for different age-based pools according to the age-based prevalence.  C. Pooling test with the same pooling size for different age-based pools according to the global prevalence. | N_test_ per case | Pooling test of separating samples by age groups is a measure that could improve the use of resources compared to the estimation of group size based on the overall prevalence of positives. | Fernandez-Salinas J, et al. (2021)[54] |
| Simulation study | General population | Pooling size | PCR | A. Individual test.  B. Routine pooling test involving heterogeneous samples.  C. Context-sensitive pooling test involving homogeneous samples. | N_test_ | The novel context-sensitive approach, which uses pooled samples from homogeneous population groups, could substantially reduce the number of tests required to screen a population. | Deckert A, et al. (2020)[53] |
| Simulation study | General population | Pooling size | PCR | A. Individual test.  B. Pooling test of the optimal pooling size considering the prevalence.  C. Pooling test of the optimal pooling size considering the prevalence and the correlation among individuals. | ·N_test_  ·N_test-saving_ | Incorporating correlation in the analysis of pool testing strategies improves the expected efficiency and broadens the settings in which the technique is preferred over individual testing. | Basso LJ, et al. (2022)[57] |
| Simulation study and real-world study | General population | Pooling size | PCR | A. Random pooling test.  B. Pooling test considering the correlation among individuals within a pool.  C. Pooling test using the social graph to form pools. | ·Ratio of number of tests. | Using the Markov modulated process model can adjust the number of pools and reduce the expected relative cost. | Lin YJ, et al. (2021)[58] |
| Simulation study | General population | Pooling size | PCR | Family-based pooling test of pooling size of 16 and 32 considering the test capacity per day of 25k and 50k. | ·N_infected_^i^ | In the event that a vaccine would not become available in due time, the use of universal testing in combination with stringent contact reductions could be considered as a strategy to eradicate the virus. | Libin PJK, et al. (2021)[56] |
| Simulation study and lab study | General population | Pooling approach | PCR | A. Dorfman pooling test.  B. Pooling test for each sample tested in multiple pools. | ·Daily detection capacity  ·Sensitivity  ·Number of sample results represented by each test  ·N_T-pos_^j^  ·N_test-saving_ | Group testing for SARS-CoV-2 can be a highly effective tool to increase surveillance coverage and capacity when resources are constrained. | Cleary B, et al. (2021)[80] |
| Simulation study | General population | Pooling size and frequency | PCR | Pooling test with pooling size of 1, 20, and the optimal pooling size with different frequencies (everyday, workday, bi-weekly, weekly, and monthly) considering the prevalence and the correlation among individuals. | ·N_test_  ·IR^k^ | Repeated testing reduces the infection probability at the time of each test and increases the efficiency of pooled testing. | Augenblick N, et al. (2022)[59] |
| Simulation study | General population | Pooling approach | PCR | A. Individual test.  B. Pooling test repeated multiple times. | ·The number of persons per test  ·The upper bound for the fraction of N_miss_^l^  ·FNR^m^ | The optimal pool size and efficiency of pooling strongly depend on the infection level of the population. Replicates help lower the pooled testing risk factor. | Hanel R, et al. (2020)[71] |
| Simulation study | General population | Pooling approach | PCR | A. Individual test.  B. Dorfman pooling test  C. Splitting pooling test: samples in the negative pools are recombined to new pooling tests and samples with two negative results are identified negative. | ·N_F-pos_  ·N_test_  ·N_F-neg_ | To lower the number of tests that must be carried out, the size of the pools can also be adjusted depending on how widespread the virus is in the community. | Litvak E, et al. (2020)[72] |
| Comparative Study and simulation study | General population | Pooling approach | PCR | A. Dorfman pooling test.  B. Sequential pooling test: the positive pool is divided into several sub-pools of pooling size of 3 and the samples in the positive sub-pool are tested individually. | ·N_test_  ·Ratio of number of tests | The size of the pool is an important parameter as it determines the total number of tests required. A proper choice of group size will yield the minimum number of tests required. | Cheng CH, et al. (2021)[66] |
| Simulation study | General population | Pooling approach | PCR | A. Random sequential pooling test the positive pool is divided into several sub-pools and the samples in the positive sub-pool are tested individually.  B. Informed sequential pooling test: divide subjects with similar risk of infection (for example, by age and sex) into the same pool. | ·N_test_ per subject | ·The sequential pooling approach is more efficient than the one-step pooling method.  ·The informed version of sequential pooling can further improve its performance, in particular for larger size pools and moderate to large virus frequency. | Millioni R, et al. (2020)[55] |
| Simulation study | General population | Pooling approach | PCR | A. Individual test.  B. Binary pooling test of the best number and depth of branches considering the prevalence. | ·Ratio of number of tests. | ·The stratification of pooling gives the flexibility to organize the turnaround time, the personnel involved, and finally the cost.  ·The dilutional effect and the probability of positive samples should be considered in the optimum strategy. | Perivolaropoulos C, et al. (2021)[67] |
| Simulation study | General population | Pooling approach | Droplet digital PCR | Nested pooling test: the positive pool is then divided into several small pools. | ·N_test_ per subject  ·Accuracy  ·Cost  ·Expected number of tests per stage  ·N_cases_^n^ (informed) | Given an upper bound of pooling size (psmax) and the prevalence (p), it is advisable to use a strategy with sequence of pool sizes (3k, …, 3) | Armendariz I, et al. (2021)[68] |
| Simulation study | General population | Pooling approach | PCR | Pooling test strategy based on optimization algorithm: the positive pool is then divided into several small pools and tested in the next stage. | The percentage of tests required compared to individual testing | The number of tests by using the pooling method is much lower than individual testing. In the case of India, the tertiary splitting procedure and a pool size of 9 are most suitable. | Rai B, et al. (2020)[69] |
| Simulation study | General population | Pooling approach and pooling size | PCR | Pooling test of pooling size of 20 in the family with different prevalence, then re-test with mini-pool for batches with positive results using pooling size of 20, 10, 5, 4, 2. | N_test_ | This pooling strategy can rapidly screen people in high-risk groups for COVID-19 infections and quarantine those who test positive. | Kei SN, el al. (2022)[70] |
| Simulation study | General population | Pooling approach | PCR | Multi-stage pooling test: 3 or more stages using the overlap strategy (some samples are detected in both pools). | ·IR  ·N_test_ | The multi-stage pooling test reduced the false-positive and false-negative results and missing samples during initial tests. A risk rate based, algorithmic guided, multiple-level, pooling strategy can be used for population screening for severe infectious disease when drugs or vaccines are not available. | Gu T, et al. (2021)[73] |
| Simulation study | General population | Whether pooling and pooling approach | PCR | A. Individual test.  B. Dorfman pooling test.  C. Matrix pooling test. | ·Costs  ·Cost per test  ·Positive rate | At extreme prevalence of 0.01% or 10%, Dorfman pooling test is the most economic, while at moderate prevalence, the matrix pooling test is the most economic. | Kim EY, et al. (2022)[74] |
| Simulation study and experimental investigation | General population | Whether pooling and pooling approach | PCR | A. Individual test.  B. Matrix pooling test.  C. OptReplica pooling teat: each patient is allocated in the first pool and replicated in another pool with the smallest number of allocated patients (an expansion of matrix pooling test). | ·N_test-saving_ | It is suggested to use the pooling technique in conjunction with an advanced replication scheme in which each patient is allocated in two or more groups to reduce the total number of tests and to allow testing of even larger numbers of people. | Žilinskas J, et al. (2021)[75] |
| Simulation study | General population | Whether pooling and pooling approach | PCR | A. Individual test.  B. Dorfman pooling test.  C. Pentagram mini-pooling test: As for the second stage of Dorfman pooling test with pooling size of ten, two samples are collected from each person in the positive pool and the twenty samples arranged in the shape of pentagram are pooled into six pools (five “three-in-one” pooled samples and one “five-in-one” pooled sample). | N_test_ | Pooling test is an effective approach for mass nucleic acid detection in regions with low infection rates (usually *p*≤0.01). When the infection rate exceeds 0.3), pooling test becomes meaningless. The Pentagram mini-pooling test increases testing efficiency by 40% compared with Dorfman pooling test with pooling size of ten. | Zhou D et al. (2022)[76] |
| Lab study and observational study | General population | Pooling approach | PCR | Subsample pooling test in the hypercube algorithm. | ·Loss of sensitivity compared to individual test  ·N_test_ per subject  ·N_infected_ | The algorithm for pooling subsamples based on the geometry of a hypercube that, at low prevalence, accurately identifies individuals infected with SARS-CoV-2 in a small number of tests and few rounds of testing, which made the cost of mass testing be reduced by a large. | Mutesa L, et al. (2021)[77] |
| Simulation study | General population | Pooling approach | PCR | Pooling test under different prevalence, edge, and dimension using the hypercubic method. | N_test_ | Every edge had a best performance range | Wu, TY, et al. (2022)[78] |
| Simulation study | General population | Pooling approach | PCR | A. Dorfman pooling test.  B. Recursive pooling test.  C. Matrix pooling test.  D. D-Optimal Pooling Experimental design (DOPE): a novel Bayesian pooling strategy. | ·FNR and FPR  ·N_test_ | DOPE drastically increased test throughput and decreased testing error rates, which is conducive to helping mitigate the current pandemic, as well as future ones. | Daon Y, et al. (2021)[79] |
| Simulation study | General population | Whether pooling and pooling approach | PCR | A. Individual test.  B. 2-stage Dorfman pooling test.  C. Binary splitting pooling test.  D. Optimized recursive binary splitting pooling test.  E. Matrix pooling test.  F. Sobel-R1: a decision tree approach based on binomial distribution. | ·Confirmed cases per test  ·Time to test the whole population  ·N_T-pos_, N_F-pos_  · N_cases_  ·Number of quarantined individuals | ·The adoption of pool-based testing strategies increased the speed and throughput of testing for SARS-CoV-2 compared to individual testing.  ·The optimal identified cases per test depend on IR.  ·Recursive binary splitting and the Sobel-R1 method would allow to significantly improve the optimal identified cases per test. | de Wolff T, et al. (2020)[81] |

^a^PCR: Polymerase chain reaction

^b^N_test_: Number of tests.

^c^N_add_: Additional number.

^d^N_test-saving_: Number of saving tests compared to individual testing.

^e^RAT: Rapid antigen test.

^f^N_F-neg_: Number of false negative results.

^g^N_F-pos_: Number of false positive results.

^h^FPR: False-positive rate.

^i^N_infected_: Number of infected people.

^j^N_T-pos_: Number of true positive results.

^k^IR: Infection rate.

^l^N_miss_: Number of missed infections.

^m^FNR: False-negative rate.

^n^N_cases_: Number of confirmed cases.
