# Supplementary figures and images for: Optimization of Screening Strategies for COVID-19: Scoping Review
Source: JMIR Public Health Surveill. 2024 Feb 27;10:e44349. doi: 10.2196/44349 (PMC10933748; doi:10.2196/44349)

**Multimedia Appendix 4**

Conceptual model for screening strategy development.


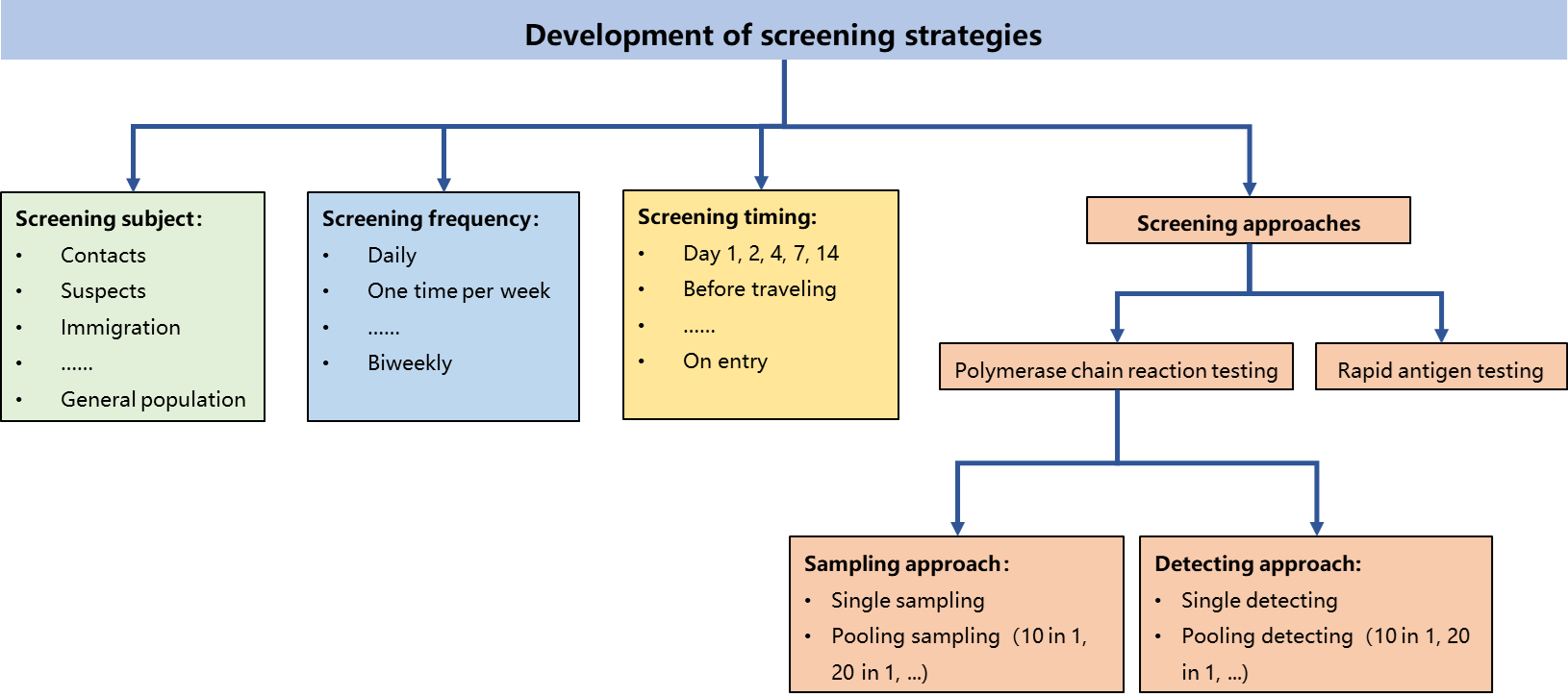

Supplement: Multimedia Appendix 4 [file publichealth_v10i1e44349_app4.docx]
